# The Impact of Social Media on Otolaryngology Literature: Analyzing the Correlation Between Altmetrics and Citation Count

**DOI:** 10.1002/oto2.70010

**Published:** 2024-09-29

**Authors:** Salman Hussain, Abdullah Almansouri, Hamad Almhanedi

**Affiliations:** ^1^ Department of Otolaryngology–Head & Neck Surgery University of Ottawa Ontario Canada; ^2^ Department of Otolaryngology–Head and Neck Surgery Jaber Alahmad Hospital Kuwait; ^3^ Department of Otolaryngology–Head & Neck Surgery McGill University Montreal Canada

**Keywords:** altmetrics, attention, citation count, education, social media

## Abstract

**Objective:**

The altmetric attention score (AAS) is an alternative metric that tracks article sharing via online platforms, reflecting an article's online attention trend. The objective of this study was to analyze the impact of social media on Otolaryngology–Head and Neck Surgery (OHNS) literature and analyze the correlation between AAS and citation count.

**Study Design and Setting:**

A retrospective review of otolaryngology journal article citation data and Altmetric attention score.

**Methods:**

The top 10 OHNS journals with highest impact factors were identified using the Journal Citation Reports (JCR). The number of citations in 2018 and 2019 were extracted from JCR and AAS was extracted from the altmetrics website. The primary outcome of this study was to establish whether a correlation between AAS and citation count exists, and whether AAS could serve as a valid alternative metric to assess the quality of individual articles.

**Results:**

By analyzing data from 3729 articles, a weak statistically significant positive correlation was identified between AAS and citation count (*r* = 0.18, *P* < .001), and between number of citations and Twitter activity (*r* = 0.18, *P* < .001). In addition, a statistically significant strong correlation was seen between Twitter activity and AAS (*r* = 0.79, *P* < .001).

**Conclusion:**

The current results clearly illustrate a weak correlation between AAS and citations and between Twitter activity and citations. Due to various limitations, the use of AAS should be limited to serve as a complementary metric to the current gold standard rather than an alternative metric.

Objective measurements that reflect the quality of scientific publications are crucial. There has been a growing body of movement towards finding the most optimal method of assessing the quality of scholarly output. Bibliometric indicators (such as the journal impact factor and h‐index) are mathematical methods used to analyze the influence and impact of scholarly output.^1^ In addition, the number of times an article has been cited in other scholarly articles (citation counts) has been considered among the most widely used indicators of assessing the impact of scientific work.^2^ In recent years, social media platforms have become an area of particular interest for the academic medical community where scholarly articles are shared, read, and discussed. For this reason, alternative metrics have been introduced with the intention of quantifying the impact of articles shared on social media platforms as well as complimenting the conventional bibliometric indicators.^3^

The Altmetric Attention Score (AAS) is an alternative metric developed in 2010, with the aim of representing the online influence of an article. It is a score that is automatically calculated by tracking article sharing via online platforms (such as blogs, news outlets and YouTube) as well as social media platforms (such as Twitter, Facebook, and Reddit).^3^ In comparison to more traditional bibliometrics such as citation counts, which might take many years to accrue, AAS is a dynamic bibliometric which allows for continuous updates, thus reflecting an article's online attention trend.^1^

There have been a number of previous studies that found a weak to moderately positive correlation between AAS and citation counts.^3^, ^4^, ^5^, ^6^ However, this relationship has not been previously assessed in otolaryngology‐head and neck surgery (OHNS) literature. Deshpande et al evaluated the relationship between Twitter mentions and citations in otolaryngology literature.^7^ However, this study took into account only one social media outlet. Due to the increased use of social media in the field of otolaryngology–head and neck surgery, we aim to evaluate the relationship between AAS and citation counts and elucidate whether AAS can be used as a complimentary tool to traditional bibliometrics.

## Methods

The top 10 OHNS journals with the highest impact factors were identified using the Journal Citation Reports (JCR). General otolaryngology journals, as well as ones with a subspecialty focus were included in the analysis. The number of citations in 2018 and 2019 were extracted from JCR and the altmetric attention score (AAS) was extracted from the altmetrics website for every article in the selected journals. Original articles as well as review articles were included in the analysis. The years 2018 and 2019 were chosen to avoid an inflated AAS as a result of the COVID‐19 pandemic and to allow adequate time for citation accrual.^8^ In addition, to evaluate the social media presence of the included journals, we searched Twitter for each of the included journals and extracted the number of followers along with the start date of the journals' Twitter account and analyzed the journal's Twitter activity as illustrated in (Table 1). Twitter activity is defined as the number of tweets, retweets and quoted tweets containing a direct link to an article.

**Table 1:** Otolaryngology–Head and Neck Surgery Journals and Their Respective Twitter Accounts

Journal	Twitter account	Age of twitter account	Number of followers
JAMA Otolaryngology–Head & Neck Surgery	@JAMAOto	14 years (Joined July 2009)	9000
International Forum of Allergy & Rhinology	@ifar_journal	4 years (joined February 2019)	640
Rhinology	@JRhinology	2 years (joined November 2021)	403
Ear and Hearing	@EandHonline	12 years (joined Oct 2011)	2361
Otolaryngology–Head & Neck Surgery	@AAOHNS	14 years (joined March 2009)	12,200
Trends in Hearing	‐	‐	‐
Clinical and Experimental Otorhinolaryngology	‐	‐	‐
Laryngoscope	@Triological	7 years (Joined July 2014)	4091
Head & Neck			
Journal of the Association for Research in Otolaryngology	@JARO_News	3 years (joined May 2020)	508

Counts and percentages were used to summarize categorical variables. Continuous variables were summarized utilizing descriptive statistics which included the mean, standard deviation, minimum, maximum, median, interquartile range (25th percentile and 75th percentile). Pearson's correlation was used to assess the association between continuous normally distributed variables. For nonnormal distributed data, spearman's correlation was utilized. The correlation coefficient was calculated to assess the association between AAS and number of citations. It was also calculated between AAS and Twitter activity (tweets, retweets and quoted tweets containing a direct link to an article) as well as between Twitter activity and number of citations. The overall coefficient was calculated in addition to the 95% confidence interval to provide an estimate of uncertainty. An *r* value between 0 and 0.19 was considered very weak, 0.2 and 0.39 as weak, 0.40 and 0.59 as moderate, 0.6 and 0.79 as strong, and 0.8 and 1 as very strong correlation. In addition, additional subanalyses were performed using articles above the 25th percentile, above the 50th percentile and above the 75th percentile for the correlation between AAS and number of citations. The statistical analysis was conducted using R (version 3.6.3).

## Results

**Figure 1:**
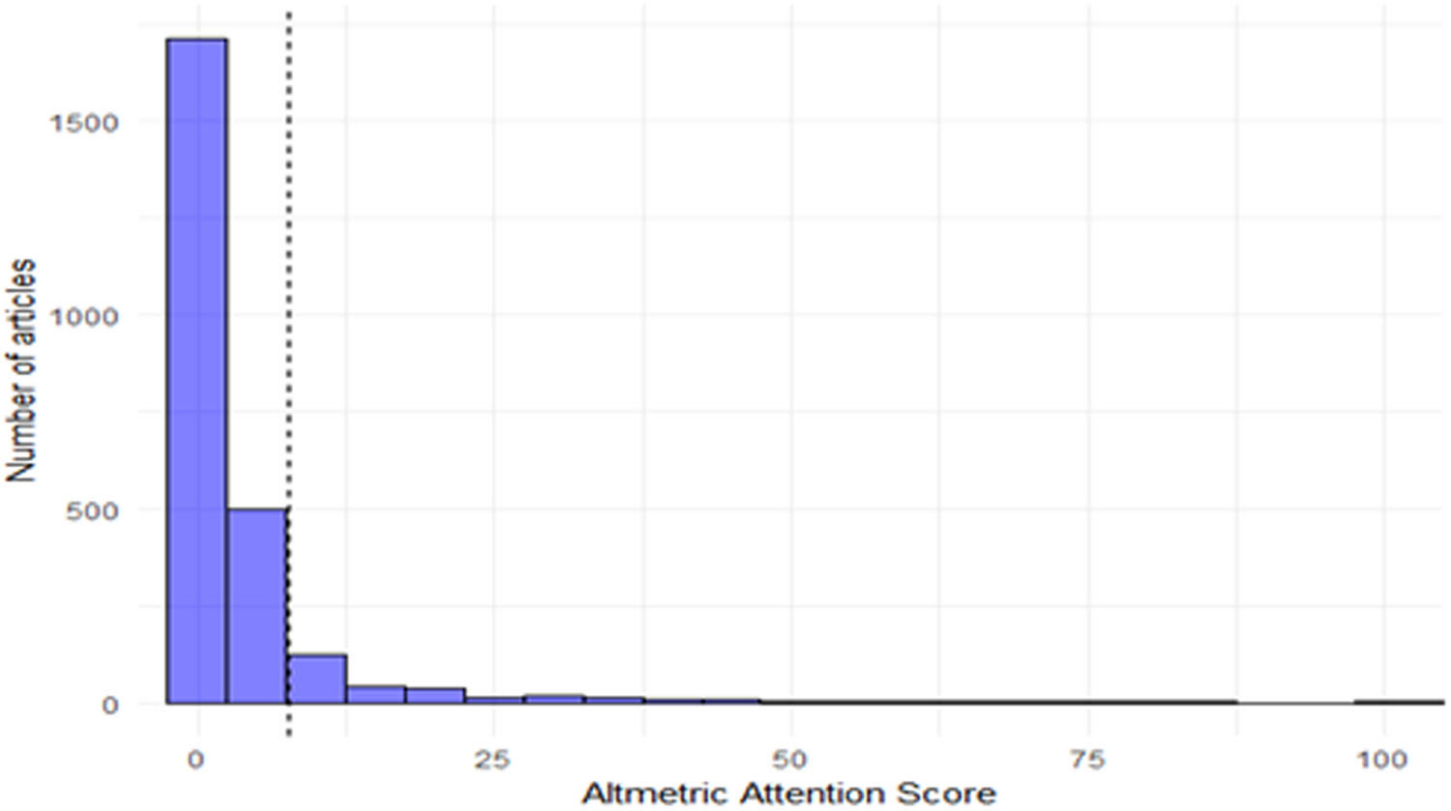
Distribution of Altmetric attention score across all journals. Figure demonstrates right skewed Altmetric Attention Score distribution.

A total of 3729 articles published in the 10 journals in 2018 and 2019 were included in the analysis (Table 2). The average AAS was 7 and the median AAS was 1 with a right‐skewed distribution. This is depicted in Figure 1, which demonstrates the distribution of AAS versus number of articles across all journals. The journal with the highest mean AAS was *JAMA‐Otolaryngology Head and Neck Surgery* (52), followed by *Trends in Hearing* (8) and *JARO‐Journal of the Association for Research in Otolaryngology* (6).

**Table 2:** Otolaryngology–Head and Neck Surgery Journals and Their 2020 Journal Impact Factors

Journal	Impact Factor 2020
JAMA Otolaryngology–Head & Neck Surgery	5.293
International Forum of Allergy & Rhinology	3.780
Rhinology	3.552
Ear and Hearing	3.377
Otolaryngology–Head & Neck Surgery	3.333
Trends in Hearing	3.156
Clinical and Experimental Otorhinolaryngology	3.138
Laryngoscope	3.131
Head & Neck	3.019
Journal of the Association for Research in Otolaryngology	3.011

**Figure 2:**
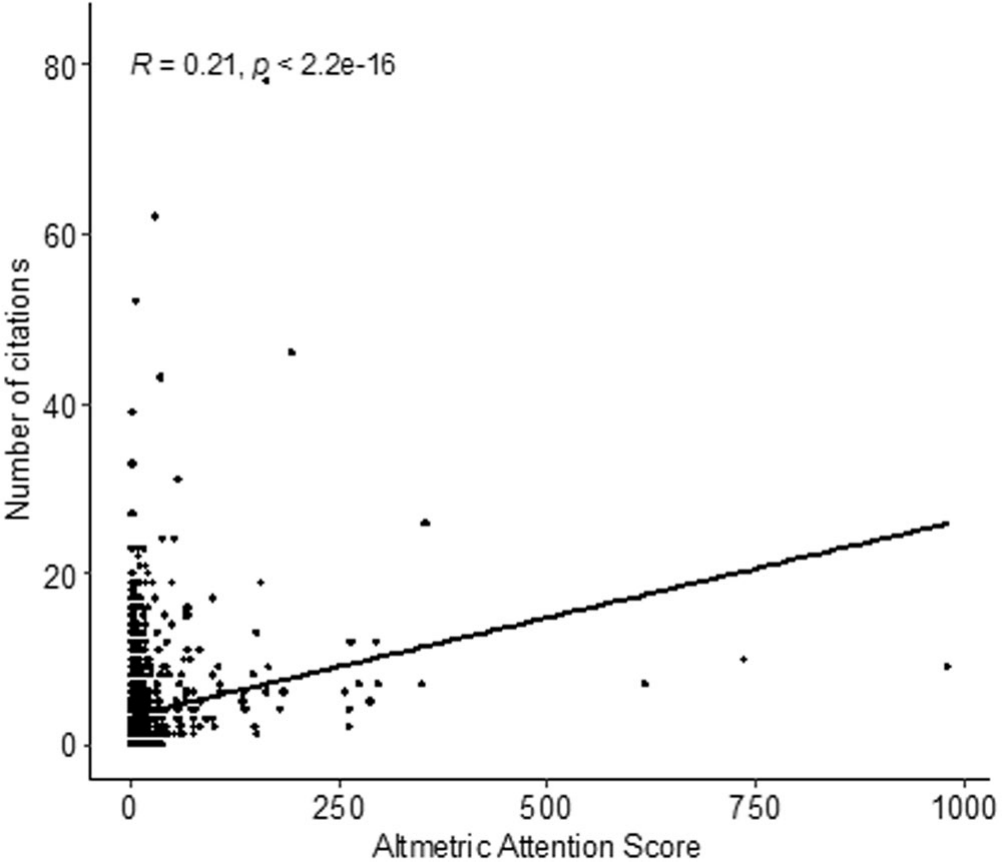
Scatter plot demonstrating correlation between Altmetric attention score and number of citations across all journals. Line represents line of best fit.

**Figure 3:**
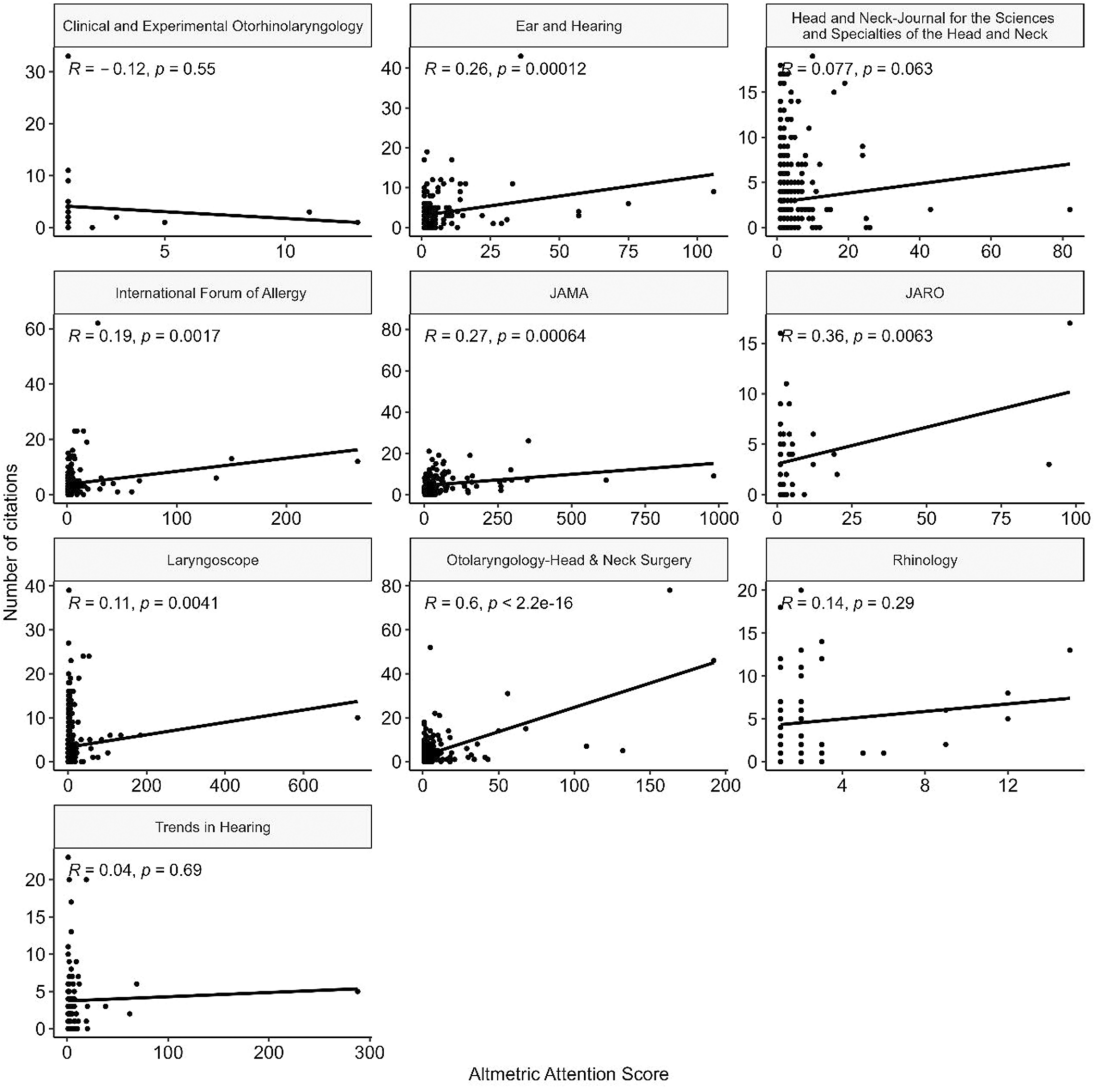
Scatter plot demonstrating correlation between Altmetric attention score and number of citations of each individual journal. Line represents line of best fit.

A very weak, statistically significant positive correlation was identified (*r* = 0.21, *P* < .001) between citations and AAS when combining the data as a whole. This is illustrated in Figure 2. The correlation of individual journals was also evaluated as demonstrated in Figure 3. A statistically significant strong correlation was noted in *Otolaryngology–Head and Neck Surgery* (*r* = 0.60, *P* < .001). On the other hand, the only journal with no correlation between AAS and citations was *Clinical and Experimental Otorhinolaryngology* (*r* = −0.12, *P* = .55). However, this correlation was statistically insignificant and only a small number of articles were included in the analysis for this journal.

When subanalyzing the articles in the top 25%, 50%, and 75% in terms of citation counts, a statistically significant very weak positive correlation between AAS and citations was observed (*r* = 0.16, 0.15, 0.09, *P* < .001 and *r* = 0.09, *P* = .05), which is similar to findings observed in the overall dataset. A similar pattern was observed when articles in the top 25%, 50% and 75% in terms of AAS were sub‐analyzed (*r* = 0.18, 0.18, 0.16, *P* < .001).

**Figure 4:**
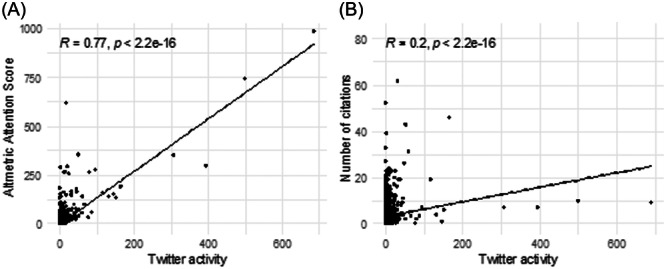
(A) Scatter plot demonstrating correlation between Altmetric attention score and twitter activity, (B) Scatter plot demonstrating correlation between number of citations and twitter activity.

The journals with the highest mean Twitter activity were *JAMA–Otolaryngology Head and Neck Surgery* (26) followed by Ear and Hearing (3.8) and *Laryngoscope* (3.6). A statistically significant very weak positive correlation was observed between number of citations and Twitter activity (*r* = 0.2, *P* < .001) and a statistically significant strong correlation was seen between Twitter activity and AAS (*r* = 0.77, *P* < .001) as demonstrated in Figure 4.

## Discussion

Our results demonstrate a statistically significant, very weak positive correlation between AAS and citation count. Upon extrapolation from our data, the lowest average AAS was 2, and the highest was 52. With regards to twitter activity, the average ranged from 1 for the journal with lowest twitter activity and impact factor and 26 for the journal with the highest twitter activity and impact factor. This shows a significant difference among the journal with the highest impact factor and lowest impact factor in the list of top 10 journals included in our study. For instance, when looking at *JAMA Otolaryngology–Head and Neck Surgery*, it had the highest average AAS of 52, in addition to the highest average Twitter activity of 26. In comparison to *Clinical and Experimental Otorhinolaryngology* which had the lowest average AAS of 2 and lowest average twitter activity of 1. This might be explained by the dominant presence of the journal on various social media platforms. Twitter activity was of a particular interest as it is considered a major platform where scientific discussions take place.^4^ These findings imply that journals that were highly interactive on various online platforms gained a great deal of attention which was reflected by a high AAS and as a result, a higher citation count. Other journals, however, were less interactive and did not have a very dominant presence on social media platforms, which resulted in a lower AAS.

By analyzing the presence of journals on Twitter, we found that *JAMA Otolaryngology–Head and Neck Surgery* had the oldest presence on Twitter and the highest number of followers. This might explain the significant differences observed in Twitter activity and AAS in comparison to other journals. In spite of having the highest average for AAS, Twitter activity and citation count, it neither demonstrated the strongest correlation between Twitter activity and citations (*r* = 0.15) nor between AAS and citations (*r* = 0.38). The strongest correlation between Twitter activity and citations was observed in *Otolaryngology–Head & Neck Surgery* (*r* = 0.52). Similarly, it demonstrated the strongest correlation between AAS and number of citations as well (*r* = 0.6). Overall, there was a very weak, statistically significant positive correlation between AAS and number of citations, and between Twitter activity and number of citations.

Our study results are consistent with findings from previous studies investigating the relationship between AAS and citations among various medical fields. Kolahi and colleagues published a meta‐analysis analyzing the correlation between AAS and citations in the field of health sciences included 35 studies from various fields and demonstrated a positive but weak correlation between AAS and number of citations. This relationship was also investigated by Asaad et al among journals in the field of plastic surgery.^6^ The study included publications in 2016 from six plastic surgery journals where they identified a statistically significant weak correlation between AAS and citation count. Similar findings were observed in multiple other fields including urology, cardiology and pediatric surgery.^5^, ^9^, ^10^ Moreover, the journal with the highest mean AAS across OHNS journals was *JAMA–Otolaryngology Head and Neck Surgery* (52). The low mean AAS is also consistently seen in the top journal of other medical specialties including Urology (67), Plastic Surgery (31), and Cardiology (31).

One of the possible explanations for the weak correlation, is that articles with a high AAS, could only mean that they intrigued the attention of the public, without necessarily having a significant scientific impact. For example, Asaad et al noted that the paper with the highest AAS was a review article about sleep wrinkles which generated an AAS of 729 but only gathered 3 citations. Similarly, Sathianathen and colleagues noted that, in the field of urology, articles with the highest AAS were those that attracted substantial online attention to the public without necessarily being scientifically impactful.^11^ Therefore, articles that garner online attention of the public or controversial articles that garner an increased online attention, will generate a high AAS without having an impact in the scientific community. This is in contrast to citation count, where articles with high citation counts generally impact the scientific community by serving as a basis for future studies.^1^ In addition, a major drawback is that AAS is a score that is generated without subjecting the article to the same rigorous review process that researchers use when citing an article. Before citing an article, the manuscript is read, critically analyzed, the results are compared and incorporated in the article being drafted in comparison to the AAS, where the score can be generated merely by the act of sharing or downloading the study.^12^ Due to these limitations, AAS is certainly not adequate to assess the quality of individual articles and is best used as a complimentary metric rather than an alternative metric to the current gold standard. Evaluation of scholarly articles should continue to be based on actual research quality including thorough methodology, replicability and validity.

To our knowledge, this is the first study to evaluate the correlation between AAS, citation count and Twitter activity among OHNS journals. The journals included in this analysis are the top ranked journals in the field. Nevertheless, this study is not without its limitations. The data pertaining to the selection of journals was obtained from one source only, the JCR 2020 (Clarivate Analytics). In addition, data from 2 years only was used to conduct the analysis in this study. Despite these limitations, the goal of the current study was to highlight the impact of social media on otolaryngology literature.

## Conclusion

A statistically significant, very weak positive correlation exists between AAS and citation count, which is in keeping with findings from other specialties. *JAMA Otolaryngology–Head and Neck Surgery* had the highest average AAS in comparison to other OHNS journals, possibly due to its ubiquitous social media presence. *Otolaryngology–Head and neck Surgery* demonstrated the strongest correlation between AAS and citation count. Due to the various limitations AAS possess, it should not be used as an alternative metric to measure scholarly impact, but rather a complimentary metric as it measures different aspects of scholarly work in comparison to current gold standard bibliometrics.

## Author Contributions

**Salman Hussain**: conception of idea, data collection, data analysis, manuscript drafting and revision; **Abdullah Almansouri**: data collection, manuscript drafting and revision; **Hamad Almhanedi**: data collection, manuscript drafting and revision.

## Disclosures

### Competing interests

None.

### Funding source

None.
